# The Structure and Reliability of the Amharic Version of the Hospital Anxiety and Depression Scale in Orphan Adolescents in Addis Ababa

**DOI:** 10.4314/ejhs.v21i1.69041

**Published:** 2011-03

**Authors:** Fentie Ambaw

**Affiliations:** Department of Health Education and Behavioral Sciences, College of Public Health and Behavioral Sciences, Jimma University. E-mail: fentambaw@yahoo.com, PO BOX: 5225, Jimma

**Keywords:** Amharic-HADS, orphan adolescents, structure of HADS

## Abstract

**Background:**

The Hospital Anxiety and Depression Scale was developed as a self-assessment tool to identify anxiety and depression in patients of age 16–65 years. Its use in younger age groups and illiterate populations is not well examined. The purpose of this study was to examine the structure, reliability, and applicability of its Amharic version in a community sample of early orphan adolescents.

**Methods:**

Secondary data primarily collected from randomly selected 804 orphans using the Amharic version of the Hospital Anxiety and Depression Scale by interview technique in March 2010 in Addis Ababa was used with permission. Confirmatory factor analysis with principal components extraction and oblique rotation (delta=0) was computed. The internal consistency of the subscales was assessed using Cronbach's alpha and the correlation between the subscales was assessed using Pearson correlation.

**Results:**

In the whole sample (age 11–18 years), two factors: anxiety and depression, explaining a total of 45.9% of the variance were found. In the 11–15 years sub-sample, the same two factors were extracted explaining a total of 45.7% of the variance. The Amharic-HADS had Cronbach's alpha of 0.81 and 0.76 in the whole sample for the anxiety and depression sub-scales, respectively. In the 11–15 years sub-sample the corresponding alpha values for anxiety and depression scales were 0.80 and 0.77, respectively. The correlation between the anxiety and the depression subscales were 0.66 (p<0.001) and 0.67 (p< 0.001) for the whole sample and for the 11–15 years group, respectively.

**Conclusion:**

Administering the Amharic version of the Hospital Anxiety and Depression Scale by interviewers gave meaningful data starting from the age of 11 suggesting successful applicability of the scale with further validation.

## Introduction

Globally, neuropsychiatric conditions are the most important causes of disability responsible for over 37% of Years of Life Lost due to disability (YLD) among people aged 15 years and older (1). In low income countries, mental disorders comprise 12% of the global disease burden. In Ethiopia, where the only source of data are health service reports and predisposing factors to emotional disturbance such as war, displacement, poverty and persistent famine exist, mental disorders contribute still for about 12% of the global disease burden (2) indicating severe underestimation.

Although researchers and other stake holders in low and middle income countries have put mental health problems specifically depression, anxiety, substance use disorders and psychosis in the first rank (3), obtaining a suitable research instrument is a major difficulty (2). In addition to that, the diagnosis and management of mental illnesses is a public health challenge (4–9) especially when clients present only with somatic symptoms (7, 10). Unfortunately, the commonest mental illnesses in primary settings: *depression* and *anxiety* (9, 11, 12, 13) present with somatic symptoms for a substantial proportion of cases (7, 8, 11, 14). Anxiety and depression may also coexist with physical illnesses worsening the symptoms, complicating the clinical presentation, minimizing response to treatments, and causing unnecessary investigation and referrals (14, 15). These challenges necessitate the use of an easy tool for screening and identifying psychiatric morbidity in primary care settings and research undertakings. The Hospital Anxiety and Depression Scales (HADS) is an instrument strongly recommended by a number of researchers for such purposes (16–21).

The HADS was developed as a self-assessment tool by Zigmond and Snaith in 1983 in England to identify anxiety and depression in non-psychiatric hospital departments in patients of age 16–65 years (14). Since then, the scale has been used successfully in a variety of situations including in primary care settings, with psychiatric patients, well person screening, community settings and antenatal clinics (16–19, 22). Although meaningful data have been obtained by interviewing illiterate clients, postal, and internet administrations (19, 23, 24), the number of studies that used these strategies are very small. Bjelland et al. (18), Herman (22), and Martin (25) in their reviews of the enormous literature on the HADS found out that the scale is easy to use and acceptable by respondents. The HADS has been designed in such a way that the scores are not affected by symptoms of somatic origin (14, 16, 19, 22) and detect anxiety and depression better than non-psychiatrist physicians (22).

Although the scale has been validated in several countries outside the United Kingdom (22), only a Nigerian study can be mentioned in Africa (19). Herman (22) has noted that the HADS scores may be different in countries with different cultural patterns of perceiving and expressing emotions. In Ethiopia, mental illnesses are very often perceived as punishments from God or possession by a devil (evil-spirit), considerably many people do not consider problems such as depression as mental illnesses (26), and the available instruments to assist clinicians as well as researchers are very few. Thus validation of such useful instruments should remain a priority.

The original developers of the test have designed it for people aged 16–65 years (14), but they did not give any explanation for not including younger adolescents. Early maturity can also be expected to exist today compared to 25 years back (the test was published in 1983) resulting in changes in the ability of younger adolescents to communicate their perceptions and emotional experiences.

The purpose of this study was to examine the structure, reliability, and applicability of interviewer administered Amharic version of the HADS (Amharic-HADS) in a community sample of orphan adolescents as young as 11 years.

## Methods

The data for this study was collected by Getachew H *et al* (27) for the purpose of comparing the level of psychological distress between AIDS and non-AIDS orphans in Addis Ababa, the capital of Ethiopia. The data was collected from 402 AIDS and similar number of non-AIDS orphans (total 804) of age 11–18 years who were registered in 16 organizations providing care and support for orphans.

Four hundred fifty four of the sample (56.5%) were females and 751 (93.4%) were in school at the time of the data collection. The 16 organizations were selected using lottery method from a total of 35 organizations providing care and support for orphans in Addis Ababa at the time of the study.

The study participants were selected randomly with proportional to size allocation from the 16 organizations. Sampling frame was prepared for AIDS and non-AIDS orphans separately to facilitate easy selection. Orphans that were apparently sick, whose parent (s) died within the last 6 months before the study, with a diagnosis of mental retardation, or with a known HIV positive status were not included in the sample. The term orphan was defined as an adolescent who has lost at least one of the parents.

The data was collected using the Amharic version of the HADS by trained high-school completed interviewers. Interviews were conducted at the offices of the organizations after informed consent was obtained from the respective organizations, guardians, and the adolescents themselves. Ethical clearance was obtained from Jimma University before data collection.

The HADS was translated into Amharic, a widely spoken language in Ethiopia, by a person who speaks both English and Amharic languages very well and knows the intent of the items. Back translation was made by another translator with similar abilities to check the accuracy of translation. The translators had a master's degree level training in counseling psychology.

SPSS for windows version 13 was used to examine the structure and the reliability of the Amharic-HADS. The suitability of the data for factor analysis was checked by inspecting the correlation matrix for presence of several coefficients greater than 0.3, finding significant (p< 0.05) Bartlett's test of sphericiy, and having the Kaiser-Meyer-Olkin (KMO) sampling adequacy index of at least 0.6.

Confirmatory factor analysis was computed as the variables were already carefully chosen by a very large number of prior studies. Principal components analysis (PCA) extraction was used to get an empirical summary of the data set as recommended by Tabachnick and Fidell for such purposes (28). Because the available literature supports that the constructs anxiety and depression are significantly overlapping, the extent to which PCA extraction inflates the estimates of variances becomes very low making the procedure appropriate. Oblique rotation with direct oblimin (delta=0) was also used because of the correlation. The number of factors to be extracted was decided using Cattles scree test as recommended by Costello AB and Osborne JW (29). Factor structures were considered fittest to the data when their item loadings are above 0.30, no or few item cross-loadings, and no factors with fewer than three items. The internal consistency of the scales were computed using Cronbach's alpha test and the correlation between the sub scales was assessed using Pearson correlation coefficients after checking the normality of distribution.

Stratified analysis was done for the whole sample and for the age group 11–15 years to find out whether the scales can be used to screen this group of adolescents. Separate analysis was not done for the 16–18 age group because the KMO index of sampling adequacy was less than 0.5.

## Results

The overall response rate in the original study was 91.8%. In the whole sample, the KMO index of sampling adequacy was 0.92 and the Bartlett's test of sphericity was highly significant (p<0.001) showing the appropriateness of the data for factor analysis. The scree plot identified two components explaining a total of 45.9% of the variance. All the items in the scale loaded clearly and strongly to one of the components except anxiety item seven (I can sit at ease and feel relaxed). This item loaded to both anxiety and depression components with loadings of 0.53 and 0.31, respectively. Another unexpected finding was that the depression item number eight (I feel as if I am slowed down) has loaded to the anxiety component clearly with a loading of 0.50.

All the original anxiety items were found to load on the anxiety component with a loading range of 0.53–0.81. All the original depression items except item number eight (I feel as if I am slowed down) were also found to load only on the depression component with a loading range of 0.41–0.78 (Table 1).

**Table 1:** Oblimin rotated PCA loadings of the HADS among a community sample of orphan adolescents (11–18 years) in Addis Ababa, March 2010

No	Items	components	

		Anxiety	Depression

13A	I get sudden feelings of panic	0.81	
3A	I get a sort of frightened feeling as if something awful is about to happen	0.73	
1A	I feel tens or wound up	0.64	
11A	I feel restless as if I have to be on the move	0.62	
5A	Worrying thoughts go through my mind	0.59	
9A	I get a sort of frightened feelings like butterflies in the stomach	0.59	
7A	I can sit at ease and feel relaxed	0.53	0.31
8D	I feel as if I am slowed down	0.50	
2D	I still enjoy the things I used to enjoy		0.78
4D	I can laugh and see the funny side of things		0.74
12D	I look forward with enjoyment to things		0.64
14D	I can enjoy a good book or radio or TV program		0.59
6D	I feel cheerful		0.47
10D	I have lost interest in my appearance		0.41
	Explained variance	37.6%	8.3%

Similar to the whole sample, in the 11–15 years sub-sample the KMO index of sampling adequacy was 0.92 and the Bartlett's test of sphericity was highly significant (p<0.001) showing the appropriateness of the data for factor analysis. Two factors were extracted explaining a total of 45.7% of the variance with clear and quite strong loadings in each component. The anxiety item number seven (I can sit at ease and feel relaxed) was found to cross-load to the anxiety and depression components with loadings of 0.48 and 0.34 respectively, and the depression item number eight (I feel as if I am slowed down) was found to load only to the anxiety component with a loading of 0.52. All the other anxiety items loaded only to the anxiety component with a loading range of 0.54 to 0.72, and all the remaining depression items loaded only to the depression component with a loading range of 0.42 to 0.86 (table 2).

**Table 2:** Oblimin rotated PCA loadings of the HADS among a community sample orphan adolescents of age 11–15 years in Addis Ababa, March 2010.

No	Items	components	

		Anxiety	Depression

13A	I get sudden feelings of panic	0.72	
9A	I get a sort of frightened feelings like butterflies in the stomach	0.71	
3A	I get a sort of frightened feeling as if something awful is about to happen	0.67	
11A	I feel restless as if I have to be on the move	0.63	
1A	I feel tens or wound up	0.55	
5A	Worrying thoughts go through my mind	0.54	
8D	I feel as if I am slowed down	0.52	
7A	I can sit at ease and feel relaxed	0.48	−0.34
2D	I still enjoy the things I used to enjoy		−0.86
4D	I can laugh and see the funny side of things		−0.81
12D	I look forward with enjoyment to things		−0.64
6D	I feel cheerful		−0.61
14D	I can enjoy a good book or radio or TV program		−0.46
10D	I have lost interest in my appearance		−0.42
	Explained variance	37.55%	8.17%

The Amharic-HADS had Cronbach's alpha value of 0.81 and 0.76 in the whole sample of orphans for the anxiety and depression sub-scales, respectively. In the 11–15 years sub-sample the corresponding alpha values for anxiety and depression scales were 0.80 and 0.77, respectively. Pearson's coefficients for the correlation between the anxiety and the depression subscales were 0.66 (p<0.001) and 0.67 (p< 0.001) for the whole sample and for the 11–15 years age group, respectively (table 3).

**Table 3:** Reliability of the Amharic HADS in orphan adolescents in Addis Ababa, March 2010.

Sample	Reliability (Cronbach's alpha)		Pearson's correlation Coefficient for anxiety versus depression scales (p-value)
	Anxiety scale	Depression scale	
11–15 years sub sample (n= 550)	0.80	0.77	0.76 (p<0.001)
Whole sample (n= 804)	0.81	0.76	0.66 (p<0.001)

In the 11–15 years sub-sample, the item-total correlation coefficients of the anxiety subscale ranged from 0.34 (for I feel restless as if I have to be on the move) to 0.63 (for I get sudden feelings of panic). This range in the whole sample was from 0.34 to 0.66 and the corresponding items forming the extreme item-total correlation coefficients were the same items as in the 11–15 years sub-sample. In both the 11–15 years sub-sample and the whole sample, deleting any item decreases the overall Cronbach's alpha value except the least correlated item (I feel restless as if I have to be on the move). The least item-total correlation coefficient of the depression subscale in the 11–15 years was 0.31 (for I have lost interest in my appearance) and the highest coefficient was 0.63 (for I still enjoy the things I used to enjoy). In the whole sample again the same items were found to make the extremes of item-total correlation coefficients from 0.29 to 0.64. In both samples, deleting any item did not improve the Cronbach's alpha value for the depression subscale (table 4).

**Table 4:** Item-total statistics of the Amharic-HADS among orphan adolescents in Addis Ababa, March 2010.

Subscale	Items	11–15 years sub sample (n= 550)		Whole sample (n=804)	

		Item-total correlation coefficient	Alpha if item deleted	Item-total correlation coefficient	Alpha if item deleted

Anxiety	I feel tens or wound up (**1A**)	0.61	0.76	0.61	0.77
subscale	I get a sort of frightened feeling as if something awful is about to happen (**3A**)	0.60	0.76	0.61	0.77
	Worrying thoughts go through my mind (**5A**)	0.53	0.78	0.52	0.79
	I can sit at ease and feel relaxed (**7A**)	0.60	0.76	0.60	0.77
	I get a sort of frightened feelings like butterflies in the stomach (**9A**)	0.46	0.79	0.47	0.79
	I feel restless as if I have to be on the move (**11A**)	0.34	0.81	0.34	0.81
	I get sudden feelings of panic (**13A**)	0.63	0.76	0.66	0.76

Depression subscale	I still enjoy the things I used to enjoy (**2D**)	0.63	0.71	0.64	0.70
	I can laugh and see the funny side of things (**4D**)	0.60	0.71	0.57	0.71
	I feel cheerful (**6D**)	0.52	0.73	0.51	0.73
	I feel as if I am slowed down (**8D**)	0.46	0.74	0.47	0.74
	I have lost interest in my appearance (**10D**)	0.31	0.76	0.29	0.77
	I look forward with enjoyment to things (**12D**)	0.56	0.72	0.59	0.71
	I can enjoy a good book or radio or TV program (**14D**)	0.34	0.76	0.32	0.76

## Discussion

The findings of this study such as the structure of the scales, the ability of the items to load to their factors, the reliability in the subscales, and the correlations between the subscales were found to agree with the findings of large scale studies in different parts of the world suggesting the potential to be used in Ethiopia. Herman (22) and Bjelland *et al* (18) reviewed over 200 articles and 747 articles, respectively and reported that most of the researchers have found out two factor structure of the HADS in different versions. Martin (25) has reported that the two factor structure of the HADS has not been true after the emergence of confirmatory factor analysis which he argues is more appropriate than exploratory factor analysis for this study. According to Martin the anxiety component of the HADS is split in to two: negative affectivity and autonomic arousal resulting in a three factor structure which means the anxiety subscale is affected by somatic illnesses. However, the two factor structure is still being reported by large scale researches that used confirmatory factor analysis (30,31). This research has used confirmatory factor analysis, as stated earlier, and identified a clear two factor structure. The new observation in this study is that the HADS can generate similar data by interviewing adolescents as young as 11 years although it was developed to be self administered for people above 15 years (14).

In terms of size of loadings, anxiety items loaded from 0.53 to 0.81 in the whole sample and from 0.54 to 0.72 in the 11–15 years age group. According to Tabachnick and Fidell (28), loadings above 0.32 are interpretable (0.32 means sharing 10% of the variance), and the above ranges fall from good to excellent. Although the anxiety item number seven (I can sit at ease and relax) has cross loaded as it has been reported by some researchers (25,30, 31), its loading to the depression factor is weak to be interpreted (28). A strange finding that the depression item number eight (I feel as if I am slowed down) has clearly and strongly loaded to the anxiety item had not been reported in any study before. Whether the difference is caused by the respondents' way of expressing their experiences or the interviewers' understanding of the intent of the item is not clear from this data.

All the other depression items loaded from 0.41 to 0.78 in the whole sample and from 0.42 to 0.86 in the 11–15 years sample which is interpreted as ranging from fair to excellent in both samples (28). Herman (22) as well as Pallant and Bailey (30) have reported that the tenth depression item (I have lost interest in my appearance) is the least loading item to the depression factor.

The internal consistencies of the anxiety subscale of the whole sample and the 11–15 years age sub-sample were 0.81 and 0.80 respectively. The corresponding alpha values for the depression subscale were 0.76 and 0.77 respectively. In Herman's review (22) the alpha value for the anxiety subscale ranged from 0.80 to 0.93 and the alpha value for the depression subscale ranged from 0.81 to 0.90. Bjelland *et al* (18) reported the alpha value for the anxiety subscale to range from 0.68 to 0.93 and the alpha value for the depression subscale to range from 0.67 to 0.93. Mykletun et al (31) found out the alpha value for the anxiety subscale to be 0.80 and the alpha value for the depression subscale to be 0.76. Although the reliability of a scale is limited to certain populations under certain conditions and cannot be conceived as a constant property of an instrument (32), this finding can be an indicator that items in the Amharic-HADS are measuring the same underlying construct similar to other versions around the world. The observed correlations between the two subscales in both samples of this study were strong similar to reports from other studies (18, 22, 33) showing the significant overlap between the two constructs.

At this point, it is very important to note that the ability of the Amharic-HADS to identify cases from non-cases cannot be concluded from this study. However, it has been shown that the Amharic—HADS was found to have a clear two-factor structure and to generate meaningful data when administered by interviewers in populations as young as 11 years suggesting its usefulness in the Ethiopian context with further validation.

## References

[1] Lopez AD, Mthers CD, Ezzati M, Jamison DT, Murray CJ, Lopez AD, Mothers CD, Ezzati M, Jamison DT, Murray CJ (2006). Measuring the global burden of diseases and risk factors, 1990–2001. Golobal burden of disease and risk factors.

[2] Alem A, Kebede D, Birhane Y, Hailemariam D, Kloos H (2006). Mental disorders. Epidemiology and ecology of health and disease in Ethiopia.

[3] Sharan P, Gallo C, Lamberte E (2009). for the World Health Organization-Global Forum for Health Research-Mental Health Research Mapping Project Group. Mental health research priorities in low-and middle-income countries of Africa, Asia, Latin America and the Caribbean. The British Journal of Psychiatry.

[4] Beljouw I, Verhaak P, Prins M, Cuijpers P, Penninix B, Bensing J (2010). Reasons and determinants for not receiving treatment for common mental disorders. Psychiatr Serv.

[5] Wittchen HU, Kessler RC, Beesdo K, Krause P, Hofler M, Hoyer J (2002). Generalized anxiety and depression in primary care: prevalence, recognition, and management. J Clin Psychiatry.

[6] Gwynn RC, McQuistion HL, McVeigh KH, Garg RK, Frieden TR, Thorpe LE (2008). Prevalence, diagnosis and treatment of depression and generalized anxiety disorders in a diverse urban community. Psychiatr Serv.

[7] Keeley RD, Smith JL, Nutting PA, Dickinson LM, Dickinson WP, Rost KM (2004). Does a depression intervention result in improved outcomes for patients presenting with physical symptoms?. J Gen Intern, Med.

[8] Bridges KW, Goldberg DP (1985). Somatic presentation of DSM III psychiatric disorders in primary care. J Psychosom Res.

[9] Wittchen HU, Hofler M, Meister W (2001). Prevalence and recognition of depressive syndromes in German primary care settings: poorly recognized and treated?. Int Clin Psychopharmacol.

[10] Munk-Jorgensen P, Rasmussen IB, Allgulander C (2006). Prevalence of generalized anxiety disorder in general practice in Denmark, Finland, Norway, and Sweden. Psychiatric Services.

[11] Conner KM (2004). Treatment of generalized anxiety disorder: current options and future developments, pp 38–41. In: Culpepper L, chair. Effective Recognition and Treatment of Generalized Anxiety Disorder in Primary Care [Academic Highlights]. Prim Care Companion J Clin Psychiatry.

[12] Mayou R, Hawton K (1986). Psychiatric disorder in the general hospital. British Journal of Psychiatry.

[13] Wittchen HU, Krause P, Hoyer J, Beesdo K, Jacobi F, Hofler M, Winter S (2001). Prevalence and correlates of generalized anxiety disorders in primary care. Fortschr Med Orig.

[14] Zigmond A S, Snaith R P (1983). The Hospital Anxiety and Depression Scale. Acta psychiatr. scand.

[15] Sareen J, Cox BJ, Clara I, Asmundson G (2005). The relationship between anxiety disorders and physical disorders in the U.S. national comorbidity survey. Depression and Anxiety.

[16] Upadhyaya AK, Stanley I (1993). Hospital anxiety and depression scale. Br J Gen Pract.

[17] Wilkinson M, Barczak P (1988). Psychiatric screening in general practice: comparison of the general health questionnaire and the hospital anxiety and depression scale. Journal of the Royal College of General Practitioners.

[18] Bjelland I, Dahl AA, Haug TT, Neckelmann D (2002). The validity of the Hospital Anxiety and Depression Scale: An updated literature review. J Psychosom Res.

[19] Abiodun OA (1994). A validity study of the hospital anxiety and depression scale in general hospital units and a community sample in Nigeria. British Journal of Psychiatry.

[20] Al-Haddad M K, Al-Garf A, Al-Jowder S, Al-Zurba FI (1999). Psychiatric morbidity in primary care.

[21] Olsson I, Mykletun A, Dahi AA (2005). The hospital anxiety and depression scale: a cross-sectional study of psychometrics and case finding abilities in general practice. BMC Psychiatry.

[22] Herrmann C (1997). International experiences with the hospital anxiety and depression scale: a review of validation data and clinical results. J Psychosom Res.

[23] Dowell AC, Biran La (1990). Problems in using the hospital anxiety and depression scale for screening patients in general practice. Br J Gen Pract.

[24] Andersson G, Kaldo-Sandstrom V, Strom L, Stromgren T (2003). Internet administration of the Hospital Anxiety and Depression Scale in a sample of tinnitus patients. J Psychosom Res.

[25] Martin C R (2005). What does the hospital anxiety and depression scale (HADS) really measure in liaison psychiatry settings?. Current Psychiatry Reviews.

[26] Deribew A, Tamirat YS (2005). How are mental health problems perceived by a community in Agaro town?. Ethiop J Health Dev.

[27] Getachew H, Abebe L, Kasahun W, Ambaw F Psychological distress and its predictors in AIDS and non-AIDS orphan adolescents in Addis Ababa city: a comparative study, 2010.

[28] Tabachnick BG, Fidell LS (2001). Using multivariate statistics.

[29] Costello AB, Osborne J W (2005). Best Practices in Exploratory Factor Analysis: Four Recommendations for Getting the Most From Your Analysis. Practical Assessment Research & Evaluation.

[30] Pallant JF, Bailey CM (2005). Assessment of the structure of the Hospital Anxiety and Depression Scale in musculoskeletal patients. Health and Quality of Life Outcomes.

[31] Mykletun A, Stordal E, Dahl A (2001). Hospital Anxiety and Depression scale: factor structure, item analysis and internal consistency in large population. British Journal of Psychiatry.

[32] Streiner DL, Norman GR (1995). Health measurement scales: A practical guide to their use and development.

[33] Crawford JR, Henry JD, Crombie C, Taylor EP (2001). Normative data for the HADS from a large non-clinical sample. British Journal of Clinical Psychology.

